# Daily weekday audit and feedback to clinicians for an inpatient intervention in obstetrics: is there sustained impact over the weekend? A secondary analysis of a prospective cohort study

**DOI:** 10.1186/s43058-021-00210-0

**Published:** 2021-09-15

**Authors:** Rebecca F. Hamm, Lisa D. Levine, Meghan Lane-Fall, Rinad Beidas

**Affiliations:** 1 Maternal and Child Health Research Center, Department of Obstetrics & Gynecology, University of Pennsylvania Perelman School of Medicine, 3400 Spruce Street, 2 Silverstein, Philadelphia, PA 19104 USA; 2 Department of Anesthesiology and Critical Care, University of Pennsylvania Perelman School of Medicine, Philadelphia, PA USA; 3 Penn Implementation Science Center at the Leonard Davis Institute (PISCE@LDI), University of Pennsylvania Perelman School of Medicine, Philadelphia, PA USA; 4 Department of Biostatistics, Epidemiology, and Informatics, University of Pennsylvania Perelman School of Medicine, Philadelphia, PA USA; 5 Department of Psychiatry, Perelman School of Medicine, University of Pennsylvania, Philadelphia, PA USA; 6 Department of Medical Ethics and Health Policy, Perelman School of Medicine, University of Pennsylvania, Philadelphia, PA USA; 7 Department of Medicine, Perelman School of Medicine, University of Pennsylvania, Philadelphia, PA USA; 8 Center for Health Incentives and Behavioral Economics, Perelman School of Medicine, University of Pennsylvania, Philadelphia, PA USA

**Keywords:** Audit and feedback, Dosage, Implementation strategy, Intervention use, Sustainability

## Abstract

**Background:**

Audit and feedback as an implementation strategy leads to small, but potentially important improvements in practice. Yet, audit and feedback is time and personnel intensive. Many interventions designed for inpatient care are meant to be utilized by care teams all days of the week, including weekends when research staff are at a minimum. We aimed to determine if audit and feedback regarding use of an evidence-based inpatient obstetric intervention performed only on weekdays could have a sustained impact over the weekend.

**Methods:**

This study was performed as a secondary analysis of a prospective cohort study examining the impact of implementation of a validated calculator that predicts the likelihood of cesarean delivery during labor induction. During the 1 year postimplementation period, Monday through Friday, a member of the study team contacted clinicians daily to provide verbal feedback. While the same clinician pool worked weekend shifts, audit and feedback did not occur on Saturdays or Sundays. The primary outcome was intervention use, defined as documentation of counseling around the cesarean risk calculator result, in the electronic health record. Intervention use was compared between those with (weekdays) and without (weekends) audit and feedback.

**Results:**

Of the 822 women meeting eligibility criteria during the postimplementation period (July 1, 2018–June 30, 2019), 651 (79.2%) were admitted on weekdays when audit and feedback was occurring and 171 (20.8%) on weekends without audit and feedback. The use of the cesarean risk calculator was recorded in 676 of 822 (82.2%) of eligible patient charts. There was no significant difference in cesarean risk calculator use overall by days when audit and feedback occurred versus days without audit and feedback (weekday admissions 82.0% vs. weekend admissions 83.0%, aOR 0.90 95% CI [0.57–1.40], *p* = 0.76). There was no significant trend in the relationship between calculator use and weekday versus weekend admission by month across the study period (*p* = 0.21).

**Conclusions:**

Daily weekday audit and feedback for implementation of an evidence-based inpatient obstetric intervention had sustained impact over the weekends. This finding may have implications for both research staffing, as well as sustainability efforts. Further research should determine the lowest effective frequency of audit and feedback to produce implementation success.

Contributions to the literature

- Audit and feedback leads to small, but potentially important improvements in professional practice. Yet, the dosage of audit and feedback for optimal implementation is unknown.
- In this secondary analysis of a large, prospective cohort study in inpatient obstetrics, we determined that daily weekday audit and feedback had a sustained impact on intervention use over the weekend.
- The finding that audit and feedback is not required on weekends may have implications for both research staffing, as well as sustainability efforts for other inpatient implementation studies.

## Background

Audit and feedback is a two-step process to improve healthcare quality. First, individuals or groups are assessed and compared either to each other or to standard targets. Second, feedback is offered to stimulate improvement. Audit and feedback leads to small, but potentially important improvements in professional practice [1–4].

Audit and feedback is time and personnel intensive. We have little understanding of how best to deliver audit and feedback [5]. Importantly, the dosage of audit and feedback for optimal implementation and sustainment is unknown [2].

Here, we evaluated audit and feedback as an implementation strategy for incorporation of a validated calculator that predicts likelihood of cesarean delivery during labor induction into inpatient obstetric care at one university-based labor unit [6, 7]. Like many inpatient care interventions, this calculator was meant to be utilized by clinical teams on a daily basis. During implementation, audit and feedback occurred on weekdays, but not on weekends. In this analysis, we aimed to determine if daily weekday audit and feedback had a sustained impact on intervention use over the weekend.

## Methods

This is a secondary analysis of a prospective cohort study of women undergoing labor induction at our institution before and after implementation of the cesarean risk calculator into usual clinical care [7]. Prior to implementation, the cesarean risk calculator was not used on our unit. The calculator was implemented on July 1, 2018, utilizing audit and feedback as the primary implementation strategy, and the postimplementation period was from July 1, 2018, to June 30, 2019. The project was approved by the University of Pennsylvania Institutional Review Board as quality improvement.

During the postimplementation period, clinicians were expected to recognize an eligible woman for use of the cesarean risk calculator during admission (undergoing a term (≥ 37 weeks) labor induction for any indication and met the following inclusion criteria: ≥ 18 years of age, singleton gestation in cephalic presentation, intact membranes, and an unfavorable cervix (Bishop score of ≤ 6 and cervical dilation ≤ 2 cm)). Women were ineligible for the intervention if they had a prior cesarean delivery, contraindication to vaginal delivery, major fetal anomaly, did not speak English, or had human immunodeficiency virus (HIV), hemolysis, elevated liver enzymes, low platelet count (HELLP) syndrome, eclampsia, or intrauterine growth restriction with abnormal umbilical artery Dopplers. The clinician would then be expected to obtain the calculator result from the online calculator, place a sticker with the predicted likelihood of cesarean next to the patient’s information on the central labor and delivery board, counsel the patient on their range of cesarean risk, and document this counseling in the Electronic Health Record (EHR).

Monday through Friday, a member of the study team reviewed all women admitted to labor and delivery since the last audit was performed who met criteria for the cesarean risk calculator and whether documentation of counseling around the cesarean risk was present in the EHR. This study team member then contacted the inpatient clinician primarily managing laboring patients (a post-graduate year 1 through 4 obstetric resident physician) daily to provide verbal feedback on calculator use. The feedback report included a review of all women admitted to labor and delivery since the last audit who met criteria for the cesarean risk calculator, with a breakdown by whether documentation of counseling around the cesarean risk was present in the EHR. The report included a recommendation to utilize the calculator for women who were still undergoing labor induction, eligible for the calculator, and did not yet have counseling around the result documented. No specific comparators, such as previous performance, were used in the report. This report gave the opportunity for clinicians (including the clinician who received the report) to increase cesarean use calculator utilization for eligible women in real-time. While the same clinician pool with the same levels of training worked weekend shifts, audit and feedback did not occur on Saturdays or Sundays. For this analysis, holidays occurring on weekdays were grouped as weekend admissions. The primary outcome of this analysis was intervention use, defined as documentation of counseling around the cesarean risk calculator result, in the EHR. Intervention use was compared between those with (weekdays) and without (weekends) audit and feedback.

Bivariate comparisons were performed with chi-square tests for categorical variables and Wilcoxon rank sum tests for continuous variables, where appropriate. Multivariable logistic regression was used to adjust for confounders. The Mantel-Haenszel test for trend was used for temporal analysis. Statistical analyses were performed with Stata 15 (StataCorp, College Station, TX). The sample size was determined by women meeting inclusion criteria for the cesarean risk calculator during the postimplementation period.

## Results

There were 822 women meeting eligibility criteria who delivered during the postimplementation period. Of the 822 included women, 651 (79.2%) were admitted on weekdays when audit and feedback was occurring and 171 (20.8%) were admitted on weekends without audit and feedback. Demographic and clinical characteristics of the study cohort by weekday versus weekend admission are detailed in Table 1. The only significant difference noted between exposure groups was that women admitted for labor induction on weekdays had less favorable cervices as determined by Bishop scores when compared to women admitted on weekends.

**Table 1:** Demographic and clinical characteristics of the post- implementation group by weekday or weekend admission. This study sample includes all patients admitted for labor induction at the Hospital of the [insert institution] from July 1, 2018, to June 30, 2019, meeting inclusion and exclusion criteria for use of the cesarean risk calculator

		Weekday ***N*** = 651 ***n*** (%)	Weekend/holiday ***N*** = 171 ***n*** (%)	***P*** value
Maternal age^a^		29 (24, 33)	29 (24, 33)	0.30
Race	*Black*	418 (64.2)	113 (66.1)	0.88
	*White*	162 (24.9)	39 (22.8)	
	*Asian*	39 (6.0)	9 (5.3)	
	*Other*	32 (4.9)	10 (5.8)	
Ethnicity	*Hispanic*	28 (4.3)	11 (6.4)	0.24
Insurance	*Private*	290 (44.5)	72 (42.1)	0.57
	*Medicaid/Medicare*	352 (54.1)	95 (55.6)	
	*Uninsured*	9 (1.4)	4 (2.3)	
Maternal BMI at last prenatal visit (mg/kg^2^) ^a^		32.4 (28.0, 37.7)	32.6 (27.3, 40.0)	0.63
Nulliparity		403 (61.9)	112 (65.5)	0.39
Gestational age ≥ 40 weeks gestation		199 (30.6)	57 (33.3)	0.49
Indication for induction				0.10
	*Postdates*	40 (6.1)	20 (11.7)	
	*Maternal indications*^b^	255 (39.2)	61 (35.7)	
	*Fetal indications*^c^	210 (32.3)	54 (31.6)	
	*Elective/other*^d^	146 (22.4)	36 (21.1)	
Modified Bishop score ^a^		2 (1, 3)	3 (2, 4)	0.02
Calculated cesarean risk	*< 20%*	214 (32.9)	52 (30.4)	0.64
	*20.39.9%*	244 (37.5)	73 (42.7)	
	*40–59.9%*	148 (22.7)	34 (19.9)	
	*≥ 60%*	45 (6.9)	12 (7.0)	

^a^Median [IQR]

^b^Examples include chronic hypertension, gestational hypertension, preeclampsia, diabetes, renal disease, history of venous thromboembolism, cardiac disease, or other chronic medical condition where induction was recommended

^c^Examples include oligohydramnios, intrauterine growth restriction, and abnormality on fetal testing

^d^Examples of “other” include history of an intrauterine fetal demise, vaginal bleeding at term, and cholestasis

**Fig. 1:**
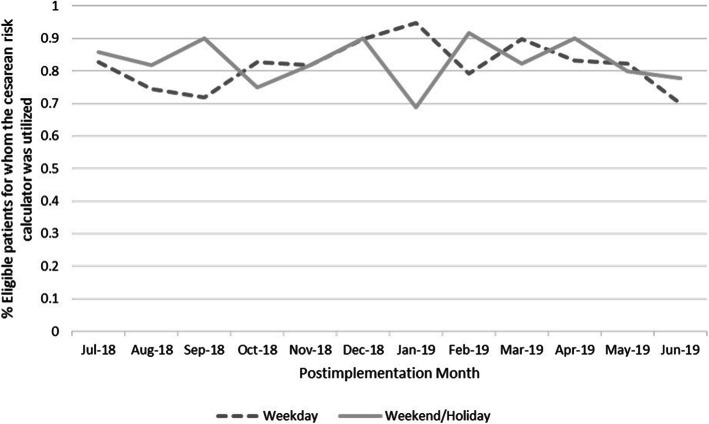
Percentage of patients for whom the cesarean risk calculator was utilized among eligible patients over the 1-year postimplementation period (July 1, 2018–June 30, 2019) stratified by weekday and weekend/holiday admissions

While the cesarean risk calculator was not used prior to implementation, calculator use was recorded in 676 of 822 (82.2%) of eligible patient charts postimplementation. There was no significant difference in cesarean risk calculator use overall by days when audit and feedback occurred versus days without audit and feedback (weekday admissions 82.0% vs. weekend admissions 83.0%, aOR 0.90 95% CI [0.57–1.40], *p* = 0.76). There was no significant trend in the relationship between calculator use and weekday versus weekend admission by month across the study period (*p* = 0.21; Fig. 1).

## Discussion

In this study, daily weekday audit and feedback for implementation of an evidence-based inpatient obstetric intervention had sustained impact over the weekends. This finding was present despite the fact that our audit and feedback method allowed for real-time improvement in intervention utilization. Additionally, this finding was stable over time.

Many patient-facing interventions designed for inpatient clinical care are meant to be used by care teams all days of the week, for many patients per day. Such interventions often require a clinician to actively remember to utilize the intervention. Audit and feedback is a common tool meant to enhance clinician adherence to practice change. Yet, for a clinician to utilize an evidence-based tool for every patient, do they need feedback daily? Weekly? Monthly? In a qualitative study evaluating how clinicians interacted with audit and feedback, participants had difficulty interpreting longitudinal feedback data, possibly indicating the benefit of more immediate and actionable feedback [8]. Multiple studies evaluating the impact of audit and feedback, often occurring as infrequently as monthly or quarterly, have shown only marginal impact on adherence to evidence-based interventions [2]. In contrast, it would be highly resource intensive and possibly unfeasible to perform audit and feedback daily on an ongoing basis for most interventions. In a systematic review evaluating audit and feedback interventions for improving test or transfusion ordering in the inpatient critical care setting, 16 studies were included with huge variation in feedback frequency, ranging from daily to quarterly. Due to great variability in the studies’ designs, there was little ability to make inferences regarding at what frequencies audit and feedback was most effective [9].

The frequency of daily weekday audit and feedback was selected in this work for two reasons. First, the effectiveness of the cesarean risk calculator on clinical outcomes was not yet proven at the start of this prospective cohort study. We aimed to reach as close to 100% utilization of our evidence-based practice as possible with the goal of demonstrating clinical effectiveness. Our method of feedback allowed for clinicians to use the cesarean risk calculator for eligible patients they may have missed, potentially leading to real-time improvement. Second, this was the most frequent, feasible dosage of audit and feedback we could perform within the resource constraints of the study.

In determining that adherence to our evidence-based practice was similar on weekends when daily audit and feedback was not utilized, we take the first step in determining the lowest effective frequency of audit and feedback for inpatient, patient-facing interventions. Future work should continue to address the dosage question, “How often should audit and feedback be administered?” The finding that audit and feedback may not be required on weekends has implications for both research staffing, as well as sustainability efforts. Well designed, comparative studies are needed to compare audit and feedback dosing frequencies for inpatient interventions in order to aid researchers, implementation scientists, and clinicians in improving care quality and adherence to evidence-based practices.

This study is limited in its generalizability; we evaluate audit and feedback for one inpatient, obstetric intervention at a single site using a postimplementation design. The same clinicians also worked weekdays and weekends. A larger number of sites with different staffing schedules may have provided different results. In addition, this study was not designed to compare frequencies of audit and feedback.

## Conclusions

These data begin to address an important question around the dosage of audit and feedback as an implementation strategy for inpatient obstetric care. Further, our work may preliminarily suggest to those in both implementation research and practice that staffing for audit and feedback for an inpatient intervention may not be needed on weekends.

## Data Availability

The datasets used and/or analyzed during the current study are available from the corresponding author on reasonable request.
